# Cancer genomic research at the crossroads: realizing the changing genetic landscape as intratumoral spatial and temporal heterogeneity becomes a confounding factor

**DOI:** 10.1186/s12935-014-0115-7

**Published:** 2014-11-12

**Authors:** Shengwen Calvin Li, Lisa May Ling Tachiki, Mustafa H Kabeer, Brent A Dethlefs, Michael J Anthony, William G Loudon

**Affiliations:** CHOC Children’s Hospital Research Institute, University of California Irvine, 1201 West La Veta Ave, Orange, CA 92868 USA; University of California Irvine School of Medicine, Irvine, CA 92697 USA; Department of Neurological Surgery, Saint Joseph Hospital, Orange, CA 92868 USA; Department of Neurological Surgery, University of California Irvine School of Medicine, Orange, CA 92862 USA; Department of Neurology, University of California Irvine School of Medicine, Irvine, CA 92697-4292 USA; Department of Biological Science, California State University, Fullerton, CA 92834 USA; Department of Pediatric Surgery, CHOC Children’s Hospital, 1201 West La Veta Ave, Orange, CA 92868 USA; Department of Surgery, University of California Irvine School of Medicine, 333 City Blvd. West, Suite 700, Orange, CA 92868 USA; Biola University, La Mirada, CA 90639 USA

## Abstract

The US National Cancer Institute (NCI) and the National Human Genome Research Institute (NHGRI) created the Cancer Genome Atlas (TCGA) Project in 2006. The TCGA’s goal was to sequence the genomes of 10,000 tumors to identify common genetic changes among different types of tumors for developing genetic-based treatments. TCGA offered great potential for cancer patients, but in reality has little impact on clinical applications. Recent reports place the past TCGA approach of testing a small tumor mass at a single time-point at a crossroads. This crossroads presents us with the conundrum of whether we should sequence more tumors or obtain multiple biopsies from each individual tumor at different time points. Sequencing more tumors with the past TCGA approach of single time-point sampling can neither capture the heterogeneity between different parts of the same tumor nor catch the heterogeneity that occurs as a function of time, error rates, and random drift. Obtaining multiple biopsies from each individual tumor presents multiple logistical and financial challenges. Here, we review current literature and rethink the utility and application of the TCGA approach. We discuss that the TCGA-led catalogue may provide insights into studying the functional significance of oncogenic genes in reference to non-cancer genetic background. Different methods to enhance identifying cancer targets, such as single cell technology, real time imaging of cancer cells with a biological global positioning system, and cross-referencing big data sets, are offered as ways to address sampling discrepancies in the face of tumor heterogeneity. We predict that TCGA landmarks may prove far more useful for cancer prevention than for cancer diagnosis and treatment when considering the effect of non-cancer genes and the normal genetic background on tumor microenvironment. Cancer prevention can be better realized once we understand how therapy affects the genetic makeup of cancer over time in a clinical setting. This may help create novel therapies for gene mutations that arise during a tumor’s evolution from the selection pressure of treatment.

## Introduction

The fight against cancer has been long and baffling. Worldwide instances of cancer increase through the years, yet cancer research is still attempting to comprehend its fundamental science and the history of hopeful medical advances is littered with disappointments. Predisposed cell populations can take decades to accumulate enough somatic mutations to transform into a malignant state with capacity for metastasis and death [1]. Earlier detection can allow intervention during states of lower tumor burden, and more importantly, with low tumor heterogeneity thereby improving treatment efficiency and thus reducing mortality. These promises allowed the conception of TCGA. The Cancer Genome Atlas (TCGA) project strives to sequence the entire genome of 10,000-tumor samples and to identify the genetic changes specific for each cancer [2,3]. These genetic changes include the inherited cancer risk alleles (germline mutations) and somatically acquired alleles (somatic mutations, including amplification and deletion of genomic DNA), as revealed by combining somatic and germline analysis [4].

**Figure 1:**
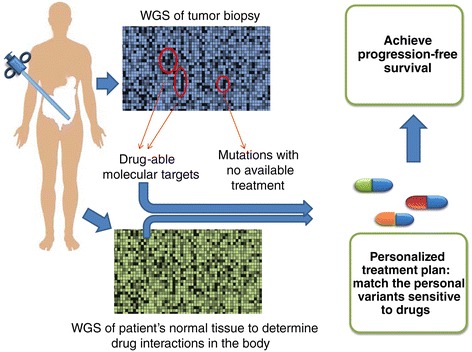
**The genome doctor’s diagnostics and treatment flow chart.** The genome sequencing comparison of the paired tissue samples of a patient’s cancer with the same patient’s normal healthy organ is thoroughly analyzed to determine the target-based and personalized profiles of cancer treatment and cancer management for a lifetime.

The TCGA is based on a hypothesis that (1) a unique and reproducible genetic difference exists among patients’ tumors, (2) the signatures can be identified utilizing whole genome sequencing (WGS) for each cancer type, and (3) as WGS technology advances, TCGA will help develop individualized treatment. Thus, the TCGA provides a hope to improve our ability to diagnose, treat, and even prevent cancer (Figure 1). The aforementioned premises were based on the notion of intertumoral heterogeneity. However, recent reports on spatial and temporal intratumoral heterogeneity [5,6] question the clinical value of TCGA sampling of a minute biopsy of tumor at a single time-point of tumor growth. This conundrum prompts the reappraisal of the validity of the TCGA approach and ponders its future utility; even though the TCGA has improved our ability to diagnose and treat a minor subset of tumors (Figure 1). Here, we review TCGA data and ponder its future utility: What point along the goal of tumor sequencing is at the crossroads? Do benefits outweigh costs of implementing new technology and new techniques?

## TCGA highlights

Breakthroughs in whole genome sequencing (WGS) of cancer were evident when recurrent mutations were found in the active site of the cytosolic NADP + −dependent isocitrate dehydrogenase 1 gene (*IDH1*), first identified in glioma [7] and then in acute myeloid leukemia (AML) [8]. All *IDH1* mutations are missense and heterogeneous with retention of a remaining wild-type *IDH1* allele; however, the *IDH1* mutants can dominantly inhibit the wild-type *IDH1* epigenetics in cells [9]. How the loss-of-function mutations for isocitrate and α-ketoglutarate interconversion relates to tumorigenesis remains uncertain [10]. The discovery of *IDH1* mutations has allowed the categorization of biological subgroups of glioblastoma [11] based on different cellular origins. Kim *et al.* recently developed a 42 probe set that divided glioblastoma patients into three prognostic groups, with one group surviving an average of 127 weeks in comparison to 47 and 52 weeks for the other two groups [12].

Offering another clinical application to the genomic approach, Verhaak and colleagues have published a framework for integrated genomic analysis that identifies clinically relevant subtypes of glioblastoma characterized by abnormalities in *PDGFRA, IDH1, EGFR,* and *NF1* [13] utilizing recurrent genomic abnormalities. Their gene expression-based molecular classification of glioblastoma multiforme (GBM) led to the classification of four subtypes – proneural, neural, classical, and mesenchymal, based on integrated multidimensional genomic data (patterns of somatic mutations and DNA copy number). Gene signatures of normal brain cell types show a strong relationship between subtypes and different neural lineages. Additionally, the response to conventional aggressive clinical therapy differs by subtype, with the greatest benefit in the classical subtype and no benefit in the proneural subtype. Our clinical study on GBM with WGS, however, shows that more heterogeneity exists than just these four subtypes exist (unpublished data).

The discovery of IDH1 mutation in GBM, performed by Johns Hopkins investigators, helped initiate the TCGA project of the National Cancer Institute/the National Human Genome Research Institute (NCI/NHGRI). The TCGA has sequenced about 8,000 cancer genomes of over 20 different types of cancers with documented genomic changes (Table 1). The TCGA intends to sequence 10,000 tumors while the ICGC (the International Cancer Genome Consortium) has a goal of 25,000 tumors. New data from the TCGA helped identify certain new subsets of cancer with potential clinical significance.

**Table 1:** **List of cancer types that are studied under TCGA**

Central Nervous System (Brain)		Head and Neck	
	Glioblastoma Multiforme		Head and Neck Squamous Cell Carcinoma
	Lower Grade Glioma		Thyroid Carcinoma
Breast		Skin	
	Breast Lobular Carcinoma		Cutaneous Melanoma
	Breast Ductal Carcinoma		
Gastrointestinal		Thoracic	
	Colorectal Adenocarcinoma		Lung Adenocarcinoma
	Stomach Adenocarcinoma		Lung Squamous Cell Carcinoma
Gynecologic		Urologic	
	Ovarian Serous Cystadenocarcinoma		Clear Cell Carcinoma
	Uterine Corpus Endometrial Carcinoma		Kidney Papillary Carcinoma
	Cervical Squamous Cell Carcinoma and Adenocarcinoma		Invasive Urothelial Bladder Cancer
Hematologic			Prostate Adenocarcinoma
	Acute Myeloid Leukemia		Chromophobe Renal Cell Carcinoma

Vogelstein and colleagues set a precedent by developing a protocol for WGS to address the complexity of intertumoral heterogeneity [14]. They sequenced the consensus coding sequences of breast and colon cancer, two of the most common tumor types affecting humans. By sorting 13,023 genes through a discovery screen, then filtering mutations through a validation screen, the authors identified 189 cancer-associated genes, the majority of which were not previously known to be mutated in tumors. Each tumor accumulates about 93 mutated genes, but only a few of these can enhance tumor progression. Surprisingly, no gene was consistently mutated in breast or colorectal cancer. The data reveals that the number of mutational events as a cancer progresses from benign to metastatic is much higher than originally hypothesized. Thus, WGS with a discovery phase screen may provide an unbiased approach to understanding cancer by revealing more details about the pathogenesis of cancer and providing new genes that have not been previously studied. Of note, the Vogelstein data in breast cancer only pertains to clonal mutations, as does most of the TCGA data.

Using mega-data computing, Alexandrov *et al.* analyzed 4,938,362 mutations from 7,042 cancers and extracted more than 20 distinct mutational signatures [15]. However, the biological process generating these genome-wide mutational signatures remains to be elucidated [16]. This argues for the need for more selective, biologically relevant screening technologies that will provide immediate information.

Zhao *et al.* determined that the *FGFR2IIIc*, changed from the normal *FGFR2IIIb* isoform, presented more mesenchymal features in clear cell renal cell carcinoma (ccRCC) tumors and was found in 90% of ccRCC cases studied [17]. Tumors found with *FGFR2IIIb* isoform were not differentiated from general ccRCCs. This subtype appears to exhibit better clinical outcomes and patient survival [17]. *FGFR2IIIc*, unique to renal cell carcinoma, may prove to be a significant negative clinical prognostic marker in the future.

Most recently, Kandoth *et al.,* have sequenced 3,281 tumors across 12 tumor types such as breast, uterus, lung, brain, head and neck, colon and rectal, bladder, and kidney cancers [18]. They identified 127 genes that had high likelihood of being driver genes and studied the prognostic value of these genes across multiple cancer types by using TCGA. The authors determined that mutations in known genes and some previously non-reported novel genes such as *BAP1*, *DNMT3A*, *FBXW7*, and *TP53*, correlated with poor prognosis; whereas mutations in *BRCA2* and *IDH1* correlated with improved prognosis [19]. Similar results were obtained for molecular signatures dividing gastric cancer into four subtypes [20]; however, prognostic prediction and potential target therapies that match the four subtypes remain to be developed and tested.

Although the TCGA project has the potential to direct the path of creating clinically relevant gene-sequencing panels, its efficacy, and utility are being challenged by new data on intratumoral heterogeneity and subclonal switching of cancer. The Vogelstein data on breast cancer pertains to clonal mutations, as does most of the TCGA data. The question of subclonally random mutations [21] (subclonal switching of cancer [22]) has never been approached in the TCGA project. The new subclonal random mutations [23] data may herald a transformation in the current TCGA practice of one small biopsy of a tumor from a patient at one time point. A single biopsy cannot help track down how tumors evolve with treatment such as subclonal switch and metastases in particular using single cell sorting device and its derived single cell genomic sequencing [24].

## Current challenges: TCGA project at a crossroads

TCGA operates on the assumptions that the WGS of a tumor sample would be exemplary of and provide insight into a broader idea of how an individual patient's disease manifests. Recent research by Gerlinger *et al.* [5] and Sottoriva *et al.* [6] reported the unexpected complexity of intratumoral heterogeneity within metastatic renal cell carcinoma and glioblastoma tumors, respectively, showing the genetic difference in space and time within the same tumor. Intratumoral heterogeneity can be defined as differences in cellular morphology, gene expression, epigenetic regulation, or phenotypic expression that develop after the initial genesis of cancer in a given patient. Previous work focused on gene expression—such as levels of growth factors, cell surface markers, hormone receptors, cell motility, and mitotic activity. However, most decisions on targeted therapies for tumors are based upon limited biopsy samples of the primary tumor at one specific time point, at the time of diagnosis using histology. Complex intratumoral heterogeneity at a genetic level suggests limitations on the use of current methods and gives hope to potential future abilities to improve the efficacy of treatments based on these new findings.

Gerlinger *et al.* performed exome sequencing, chromosome aberration analysis, and ploidy profiling on multiple spatially distinct sites from primary and associated metastatic renal carcinomas [5]. With a broader scope of genes to examine, this study synthesizes a personalized history of branching evolution of the cancer within each patient [25]. They discovered convergent tumor evolution since a given tumor contained different mutations of the same genes, *SETD2, KDM5C* and *PTEN*, depending on its spatial orientation [5]. Distinct mutations in the same tumor illustrate how gene expression is dynamic, providing evolutionary means for tumor cells to respond to selection pressure, changing their gene function, and adapting to the tumor environment. Alterations in epigenetic mechanisms and signaling pathways as the tumor progresses and evolves provide additional diversity generally enhancing tumor survival [25,26].

In the analysis of glioblastoma, branching evolution within brain tumors was determined by Sottoriva examining the copy number alterations of samples that had been collected from spatially distinct regions of the surgically resected tumor [6]. Copy number alterations in *EGFR, CDKN2A/B/p14ARF* were considered early events because all tumor samples shared these changes. In contrast, mutations in *PDGFRA* and *PTEN* developed later in tumor progression, finding that not all biopsy tumor fragments expressed these aberrations [6]. Similarly to the work performed by Gerlinger and colleagues, Sottoriva *et al.* generated a map of branching evolution that elucidated the origin of genetically distinct clones, over time and space. Of significance, six out of ten patients in Sottoriva’s study had tumors with regions that belonged to two or more glioblastoma clinical subtypes, suggesting that tumors contain cell populations that display varying survival outcomes and treatment responses.

Along the same line, primary triple negative breast cancers (TNBCs) have been grouped together under one name as if they share commonalties in genetic and chemotherapy treatment responses [27]. However, Aparicio and colleagues completed deep sequencing of 104 primary TNBCs; and found a wide and continuous spectrum of genomic evolution with multiple clonal frequency modes within each tumor, demonstrating intratumoral heterogeneity [28]. These reports pointed out the necessity of re-evaluating our clinical approaches to cancer treatment. Gerlinger’s and Sottoriva’s findings are expected given that a central prediction of the concept results in a mutated phenotype, the concept that stretches back more than 30 years across different cancer types [29-33]. However, these mutation based tumorigenesis and cancer progression remain to be further elucidated. Recently, scientists have developed powerful new tools such as single cell technology and biological global positioning systems [34] for studying these changes.

### Comparison of primary to metastatic cancer provides insight into cancer progression

Tumor is characterized by grade (defined by benign and malignant) and stage (local, invasive, and metastatic). WGS revealed that using primary tumor biopsies to categorize cancer subtypes might not provide an optimal strategy for adequately defining the pathological grade of a given tumor in treating the disease, as current strategies may fail to address intratumoral heterogeneity, and failing to capture right information that makes tumor resistant to therapy. In fact, primary tumor biopsies bare little resemblance to their metastatic counterparts. Sottoriva *et al.* suggest a multiple sampling scheme to determine a patient’s tumor heterogeneity before making treatment decisions [6]. However, spatially distinct tumor samples may prove difficult to obtain clinically, and multiple metastatic sampling often proves unattainable due to unacceptable risks to the patient [35]. In addition to the barrier of physically obtaining multiple regional primary and metastatic cancer samples, increased numbers of samples needed to determine heterogeneity and lineage of evolution would vastly increase the cost of cancer diagnosis and treatment planning. Responding to this tissue pressure, Marusyk and Polyak suggest investing more time in research to determine if the clonal heterogeneity of primary tumors is predictive of the clonal variances found in metastatic tumors [36].

New findings based on information obtained from multiple samplings of tumor varied spatially and temporally, differing from the previous tumor sampling method of single location at a single time, showed that multiple subtypes exist within one tumor. Indeed, within a tumor, multiple subclones (subtypes) co-exist with different levels of potential malignant behavior grades, also existing as dominant or dormant stage [22,37]. Dormant subclones may co-exist with dominate subclones; however, these two types of subclones can switch roles during tumor progression, suggesting a novel strategy may be to block the switching signaling between dominant subclones and dormant clones [22]. Specific mechanism remains to be elucidated.

**Figure 2:**
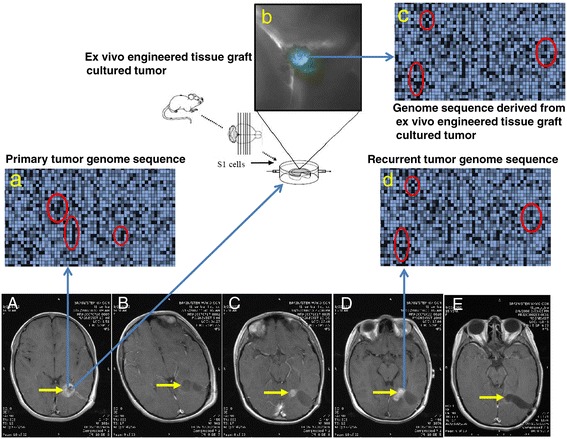
**Co-evolution of tumor (primary versus recurrent) with therapeutic-modified microenvironment in-patient in parallel with an** ***ex vivo*** **engineered tissue graft cultured tumor for time-dependent changes.** MRI images show that tumor diagnostics, resection, recurrence and yet another resection of a young female glioblastoma patient during treatment of surgery, radiation, and chemotherapy. (Yellow arrows: Tumor mass). **A**: pre-operation (post-biopsy), axial view showing diagnostics, and appearance of the tumor. **B**: Immediate postsurgery, axial view showing removal of the tumor. **C**: 3-month post-surgery, axial view showing recurrence of the tumor. **D**: 4-month post-surgery, axial view showing the growth of the recurrent tumor. **E**: second post-surgery and gamma knife, axial view showing removal of the tumor. Note that the genome sequence **(a)** obtained from the primary tumor **(A)** is compared with the genome sequence **(d)** obtained from the recurrent tumor **(D)** to determine the genetic mutations evolved under the therapeutic pressure. The genome sequence **(d)** obtained from the recurrent tumor **(D)** is compared with the genome sequence **(c)** derived from an ex vivo engineered tissue graft [91,99]-cultured tumor **(b)** to determine if the ex vivo models the genetic and epigenetic changes in-patient. Such a correlation, if established, can be used to predict the therapeutic-driven changes of intratumor spatial and temporal genetic and epigenetic information. Note that a mouse-derived ex vivo model may be replaced by using a patient-specific engineered tissue graft to screen for personalized treatment.

As cancer is an evolving target, we need to develop a dynamic model to study how a tumor evolves under therapeutic intervention (Figure 2). Also, it will be useful to study how therapeutic selection pressures a dormant subclone of a tumor to become a dominant subclone [22]. This “switch” signal affecting the dormant subclone may be derived from inflammation caused by surgery or by cellular death from chemotherapy and radiation therapy [38,39]. It may prove important to define what keeps dormant subclones from proliferating, and then what activates these cells [40].

Current concepts of cancer grade (benign, malignant) and cancer stage (local, invasive, and metastatic) may not be relevant in cancer treatment since it persists as a continuum of evolving cell populations. Lack of models for studying this continuum hinders the development of therapy and protocols to confront the changing clonal subtypes within a given tumor over time. A therapy that starts with certain tumor subtypes needs to be modified over time to address the fact that the target changes over the course of treatment. Until we get to the basic commonality of a given tumor, based upon where it initiated and whether or not all given subtype mutations share that commonality, we are only treating one type of malignant population cells where others escape therapy resulting in poor outcomes. Thus, “treating cancer by chasing mutation after mutation with expensive drug after drug is not a sustainable model — not the least because few cancers other than leukemia have simple, known genetic causes” [38].

Sonnenschein and Soto criticized the current theoretical paradigm that focuses heavily on cancer genes [41] as not every mutation in every cancer cell can be therapeutically addressed. Dr. Sangeeta Bhatia likens the diversity of cancer to HIV and HCV, highly mutable viruses, with many quasi-species existing in the body without being recognized. Current schemes of antiviral regimens can ascertain the top three forms of drug resistance and provide combination therapy to address those three only [35]. Likewise, using a drug to target one mutated genetic marker and to shut down this abnormal pathway changes the regulatory network of the cell [42]. Many cells are killed by this drug, but those that do not die and adapt to the new landscape, gives them a chance to survive, induce mutations, and become further resistant to the drug [42].

Many of the newly identified mutations do not suggest targeted therapies. Even if a tumor presents with eight to ten genetic mutations, the most important priorities would be to limit chemotherapy targets to the top three in order to limit drug toxicity and treat the most malignant, invasive subclones. Current capabilities potentially target one or two genetic markers that are shared by many patients exhibited by different types of tumors, a non-unique treatment that hardly qualifies as personalized therapy [43]. Irwin Nash thought “such genetically based personalized oncology care” is not qualified to be personal [43].

Despite possible intratumoral heterogeneity, clinical benefit has been demonstrated for the targeted therapy guided by genomic information obtained from primary bulk tumor. For example, the Biomarker-integrated Approaches of Targeted Therapy for Lung Cancer Elimination (BATTLE) clinical trial [44-46] incorporates concurrent improvements in new therapeutics with its matching indicative test [47], and recognizes that patients are destined to benefit from next-generation sequencing (NGS) approach. BATTLE’s success is likely an exceptional case, unlikely applicable to other patients due to its complicated procedure and financial cost. The concept of “actionable” somatic genomic alterations present in each tumor (e.g., point mutations, small insertions/deletions, and copy-number alterations that direct therapeutic options, obtained from systematic genomic profiling) has rarely been achieved beyond a limited number of oncogene point mutations, such as EGFR, KRAS, TP53, PIK3CA that are commonly shared by many patients and across many tumor types [48]. In fact, it is not clear that the most important priority is to target the most prevalent “actionable” mutations. This is exactly the opposite of the data reported by Beckman; his data indicate that the best approach would be to target minor resistant subclones first [49].

These studies show significant heterogeneity within a primary tumor. Single biopsies have an assumed homogeneity throughout a given tumor and its metastasis yet we know this to be false. Neither does it extend to other patients and the associated metastases because they are genetically different. Current diagnostic practice involves tumor-sampling bias. Current TCGA genomic analyses based on a single tumor biopsy yields an inaccurate and incomplete picture of the complexity of disease in a given individual. This leads to ineffective therapies [6]. The TCGA methodology may help certain patients; however, all of these discrepancies have placed the TCGA approach at a crossroads on the path toward cancer management.

This crossroads is represented by the dilemma which exists between sequencing more single-time tumor sampling of different types and sequencing multiple biopsies from different regions of the same tumor at many time points. The latter approach sounds excellent, but at what cost to reveal the genetic evolution of a tumor? It may not be technically possible. The crossroads manifest two ways of obtaining related but diverse/unique information. One way is to sequence more tumors with the past TCGA methodology of single time-point, but this way cannot determine the heterogeneity of the inside of a tumor site, the difference between diverse sites of the same tumor, nor the heterogeneity generated as a function of time, lapse rates, and irregular genetic drift. The other way of acquiring multiple biopsies from every individual tumor can illustrate an evolutionary genetic change within a tumor, but this way still has limitations of tumor sampling and the cost of sequencing. A choice on the paths for either sequencing multiple tumor types or sequencing multiple biopsies must be made, a choice with economic impact, a choice that leads to cancer prevention, a choice that is related to cancer patient’s life expectancy.

## Emerging opportunities

**Figure 3:**
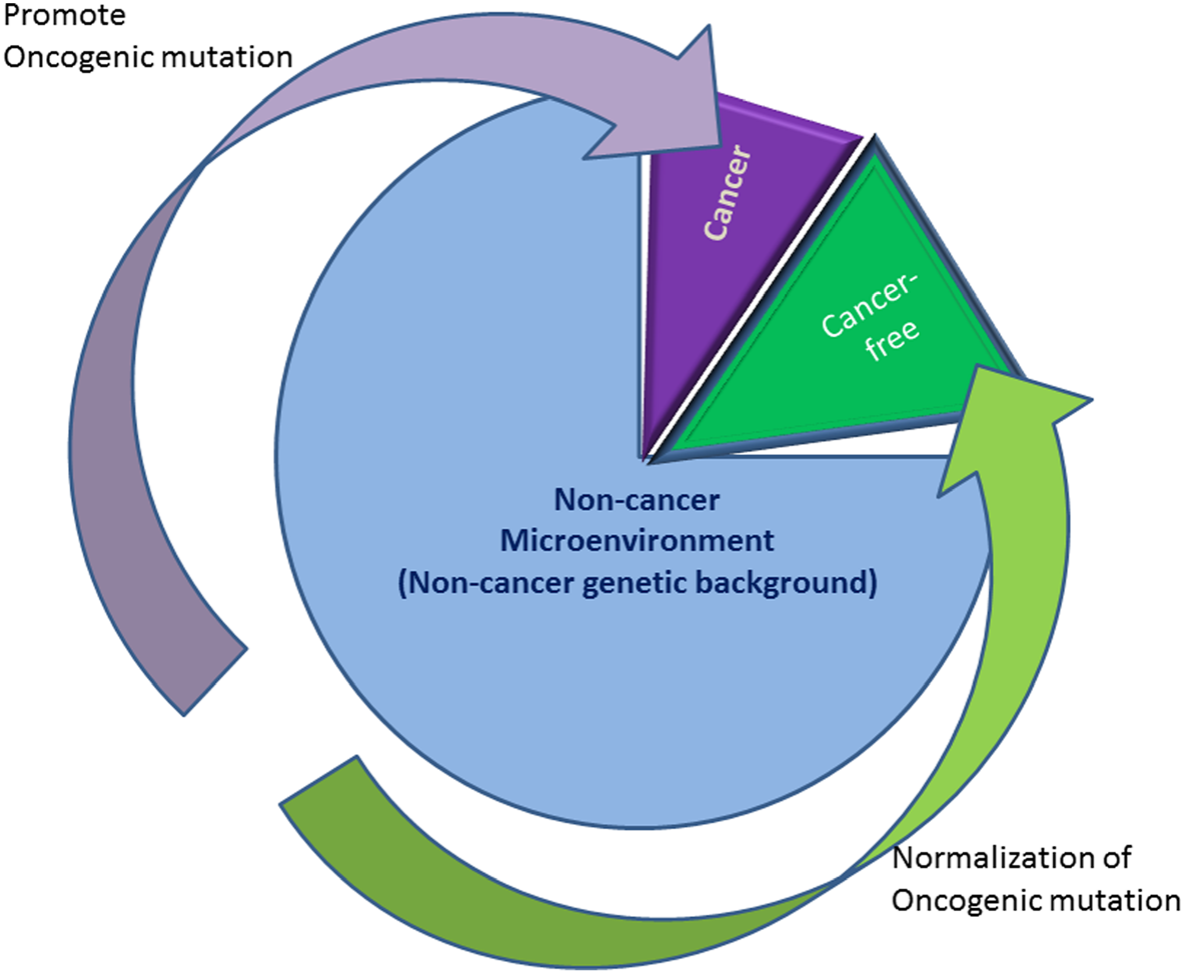
**Effects of non-cancer genetic background (cancer microenvironment) on cancer initiation and progression.** Certain patients (5% of population, e.g., Li-Fraumeni syndrome, p53, Retinoblastoma) always acquire cancer and certain patients (5% of population are cancer-free) exclude cancer. The rest 90% of population can undergo either cancer initiation or cancer-free depending on their non-genetic background because oncogenic mutation (cancer variant carriers) may not be sufficient to drive cancer initiation. The non-genetic background determines that certain patients are susceptible to cancer risk factors such as smoking, HPV, UV, food addiction, heavy metals, free radicals; these risk factors may initiate cancer for vulnerable patients. Management of these risk factors (related non-cancer genetic background) may either promote cancer or suppress cancer initiation – whole genome sequencing can help predict these risk factors thereby preventing cancer – the prevention can benefit the rest 90% population.

Recent reports point out that current TCGA projects beget the same, major problem of intratumoral heterogeneity with spatial and temporal changes. How can we overcome this problem of intratumoral heterogeneity? What are ongoing technical developments that may tackle the intratumoral heterogeneity problem? What are practical approaches to this problem based on novel technologies? What are current technical hurdles for these novel technologies (massive parallel sequencing of DNA/RNA isolated from more homogeneous cancer cell population obtained by microdissection tools)? The TCGA provides emerging opportunities for developing an integral component of diagnostics for cancer related to non-cancer genetic background (Figure 3). These include standardization of single cancer cell isolation, cancer genome sequencing devices, and development of further studies on TCGA-catalogue-guided tumoriginesis and therapy-driven cancer evolution.

### Practical approaches to intratumoral heterogeneity based on novel single cell technologies and standardization

#### Single cell analysis

To track those dominant and dormant subclones within a tumor, single cell technology has been proposed to understand and predict intratumoral heterogeneity. Not only is it necessary to determine the range of genetic markers of disease and its progression within a tumor, but we also need to study the combinations and interactions among gene mutations of subclonal populations. By understanding the genetic composition on a single-cell level, we will have greater insight into the interplay between genetic mutations within subclonal tumor populations. For example, new single cell genome technology reveals host-tumor immune interface as a key part of the glioblastoma ecosystem composed of cancer and immune cells, leading to a novel discovery suitable for target therapy [50]. Ideally, single cell genome technology would ultimately apply to other tumor ecosystems as well.

TCGA-integrated single cell analysis technology may help to uncover the role of each subclone in intratumoral heterogeneity and to understand the advantages this arrangement affords to a tumor. There are different theories of intratumoral heterogeneity, including variant subclones interacting competitively for resources versus cancer growth stemming from clonal relationships of commensalism and mutualism [36]. It appears that heterogeneity contributes to therapeutic resistance. If the potential mutability of each cancer cell can be determined and the mutation predicted, novel strategies and therapies may arise [51]. For instance, directing drugs to subclones with angiogenic properties may eradicate non-angiogenic, “free rider” subclones [36].

Current technologies for detecting single cancer cells in peripheral blood or cerebrospinal fluid, termed circulating tumor cells, represents a novel method to determine efficacy of therapeutic drugs on cancer as well as identifying tumor progression. Research has shown that identifying circulating tumor cells may predict overall survival in metastatic breast, prostate, and colorectal cancer and may provide prognoses in additional cancers, such as small cell lung cancer and hepatocellular carcinoma [52-56].

While there is significant research in the field of single cell cancer detection, CellSearch™ was the first to obtain FDA clearance for a method of detecting circulating tumor cells in patients. It uses antibody-coated magnetic beads to bind to and sort cells derived from blood or CSF samples as a means to identify cancer cells [57]. Newer mechanisms are emerging due to the rising interest in single cell classification, such as capturing cancer cells in blood utilizing a microfluidic approach, where a blood sample flows through microchannels and cells are sorted by size to identify cancer cells [58]. Most commonly, though, single cell detection uses biomarkers, such as epithelial adhesion molecule, prostate-specific membrane antigen, and cytokeratin [59]. Complete sequencing of the cancer genome will provide insight on new biomarkers that may enhance specificity in discriminating tumor cells from normal circulating cells. Furthermore, sequencing of copy number variations, SNPs, DNA methylation, and microRNA profiling from TCGA will provide information beyond recognizing gene expression for enhancing single cell detection. While immense progress has been made at a single-cell detection level, the significance of circulating tumor cells and their potential for contributing to recurrence and metastasis has yet to be fully determined [35]. Therefore, technology that amplifies the whole genome from a single cell may prove useful to advance the specificity of detecting circulating tumor cells and in further defining the role of single cells in predicting resistance to treatment or recurrence of disease.

The next step in progression of single cell analysis is massive parallel sequencing, or next-generation sequencing (NGS), which is becoming more feasible with competition to improve technology among biotech companies. Current NGS platforms, such as Illumina HiSeq2000, can allow profiling of 200 single cells in one run [60]. Implications of current platforms include being able to sample multiple sites of fluid with the potential to identify circulating cancer cells, identifying tumor cell lineage relationships, and classifying different subclones within a tumor sample at once. Barriers to massive parallel sequencing currently include high costs, which are soon predicted to decrease with rapidly advancing technology, and the necessity for high-fidelity methods of whole genome amplification of single-cell DNA without incorrect SNPs, without uneven sequencing coverage, and without allele dropout [61].

Another diagnostic tool becoming increasingly common in cancer centers is gene-sequencing panels. In March 2013, the first multi-gene DNA-sequencing tests were administered to patients through the National Health Service (NHS) in the United Kingdom to classify oncologic genetic mutations. These were designed to help physicians choose the most effective therapeutic targets for each patient’s tumor [62]. While a single genetic screen on a tumor previously cost £150 through the NHS, this 46-gene panel costs £300 [63]. This relatively inexpensive multi-gene panel aims to eradicate guesswork for selection of chemotherapy strategies, which improve efficacy by minimizing the negative consequences of ineffective treatment. Similarly, in the U.S., a genetic panel predicting prognosis, the Onco*type* DX Colon Cancer test, provides prognostic information that other diagnostic tools have not yielded; the test distinguishes the absolute increase in recurrence risk at three years between low and high-risk patients by 10% [64,65].

#### Standardization

Tools for diagnosis of cancer should be sensitive, minimally invasive, reproducible, standardized, and potentially be able to prognosticate outcomes at early stages of a disease. Researchers can collaborate with health-care groups to establish regulations for sharing genetic information from large research endeavors, like TCGA, without compromising medical ethics and patient privacy. By creating a framework for institutions to aggregate and exchange genomic data, researchers and medical providers can advance the progress of diagnosis and treatment of certain malignancies. In regards to cancer, mega-databases of thousands of tumor samples may enable faster development of TCGA catalogues for tumor progression and drug responding profiles, a routine testing like a blood biochemical profile.

The TCGA project inspires the development of TCGA-integrated instrumentation to bring down the cost and facilitate broad clinical access [66]. For example, Cancer Research UK has embedded de-identified breast cancer genetic data into a new Smartphone program so that average participants can identify copy number variations within chromosomes that are difficult to visualize. Along the same vein, emerging smartphone applications to encrypt digitized genome data will provide patients with risk factors for cancer. A third venue to utilize the TCGA with advancing technology is to create diagnostic kits for every day clinical diagnosis. Although currently in the research phase, kits, such as RightOn Cancer Sequencing Kit may be used every day in the hospital. Its technology enables identification of 1,000 cancer genes with a single test. An ideal diagnostic kit would include comprehensive profiling of all cancer types, with vast coverage, high specificity and high sensitivity to detect common and rare genetic variants, in one-cost efficient test [67]. The Ion Proton, a desktop-sized, semi-conductor based gene sequencer, can help sequence the entire human genome for less than $1,000 in two hours [66]. With the advent of multi-gene sequencing panels and portable DNA sequencers, a patient’s genetic profile will become part of the routine regimen for cancer diagnosis and treatment protocol decisions.

Can all of these diversified instruments, analytical tools, and different institutions produce the accuracy of DNA sequencing? This is particularly critical for DNA sequencing with respect to some of the personally held instrumentation as mentioned. A general guideline for the quality control of DNA sequencing should be implemented to produce medically meaningful genetic information, a regulation that should be generated by a government agency like the US Food and Drug Administration (FDA).

#### Tumorigenesis, cancer progression, and metastasis

The TCGA has shown that the mutational landscape of cancer is complex and multifaceted. In order to carry out diagnosis and treatment of an individual with potentially 500 oncogenic mutations [38], we must have an understanding of cancer initiation and progression. New algorithms (Dendrix™) have scaled up to whole-genome analysis of thousands of patients for larger data sets of TCGA for specific “driver” mutations [68]. While the number of genetic mutations in a tumor may range from 30–200 depending on the type of tumor, research has shown that approximately 2–8 of these mutations are “driver genes” [69]. A “driver gene” mutation is a mutation that provides the cancer with a small, but selective growth advantage over the surrounding cells, potentially enabling that cell to become a clone [14]. Multiple insults in these “driver genes” occur over years within a cell before the cell takes on a cancer phenotype. Passenger gene mutations, on the other hand, provide neither a positive nor a negative effect to cancer cell growth.

Cancer mutations follow a natural selection theory. Thus, when a cancer cell divides, it will acquire new mutations upon selection pressure, in addition to or altering its “driver gene mutations” [70]. These new mutations cause the new cell’s genetic composition to be slightly different from its progenitor cell. Therefore, it is not surprising that heterogeneity exists within a tumor; cells at different ends of the tumor may be genetically different. The same rules of evolution apply in metastatic cancer. Research performed by Gerlinger *et al.* showed that tumor samples from multiple primary tumor sites, perinephric fat metastasis, chest-wall metastases, and germline DNA could be synthesized into a phylogenetic tree, much in the same way that trees are constructed in the evolution of species [5]. This means metastatic tumor samples and the primary tumor itself exhibit different genetic compositions; their mutations diverged from the common mutations of the original primary tumor. The diverging genetics of metastatic tumors also stresses the importance of early diagnosis.

The evolutionary growth of cancer sounds impossible to tackle, but Vogelstein *et al.* have organized all of the known driver genes into 12 cancer cell-signaling pathways: *RAS, PI3K, STAT, MAPK, TGF-β*, DNA damage control genes, transcriptional regulation, chromatin modification, *APC, HH, NOTCH*, and cell cycle/apoptosis [14]. Rather than focusing on the differences among tumor cells, we must target the common mutations that occur before the branching, or diverging, points. Indeed, a TCGA-guided new approach to therapy has surfaced based on a comprehensive molecular analysis of tumor samples from 825 patients with breast cancer [71]. Previously breast cancers were classified in four main molecular subtypes of the disease: basal-like; luminal A and luminal B, which are both estrogen receptor (*ER*) positive; and *HER2* enriched. The TCGA analysis uncovered new mutated genes, expanding these four subtypes. For example, they found at least two subtypes of clinical *HER2*-positive tumors. One type is *ER* negative and has high levels of *EGF* receptor and *HER2* enriched in *HER2* protein phosphorylation. The other with *ER* positive shows lower DNA amplification and protein-based signaling, resembling the luminal subtypes. This may explain why current *HER2* (trastuzumab)-based treatment failed half of patients with *HER2*-positive tumors.

In certain breast cancers, mutations of genetic regulatory sequences promote cancer. Mutations such as duplications of the densely estrogen receptor-α-bound distant estrogen response elements in the chromosomal sequences 17q23 and 20q13 predict poorer outcomes and anti-estrogen resistance in patients [72].

Other researchers think that TCGA provides only part of the picture of tumor heterogeneity under pressure from drug therapy. Joan Brugge pointed out that cells that are not intrinsically resistant to a drug rewire their gene circuitry during treatment to become resistant without any genetic changes [38]. Mina Bissell and Jacqueline Lees show that tumors cannot thrive without certain signaling patterns from their neighboring cells since traditional drug screening missed that microenvironment [38]. These wake-up cells switch back on by taking advantage of interactions with normal surrounding cells [38,39]. Thus, drugs that suppress this crosstalk could prevent them from restarting a tumor after therapy [38].

TCGA genomic data has been collected simultaneously while other comprehensive “omic” profiles have begun to build extensive libraries as well in order to provide better indicators on how to holistically identify and characterize disease. The notion of calling diseases by body part is rooted in mid-1800s in France and is likely characterized by pathways and signals at the molecular level (David Agus) [66]. The importance of copy number analysis, for example, argues that tumors can be classified in those driven by either mutations (M class) or copy number aberrations (C class) (Paul C. Boutros, 11JUN2014, webinar.sciencemag.org). C class tumors include breast, ovarian, squamous cell lung, and prostate cancer. However, next generation sequencing technologies have limited ability to detect clinically relevant lower level amplifications, copy neutral loss of heterozygosity, and homozygous deletions, even at significant depth of coverage.

TCGA-integrated biochemical assays would enable monitoring of tumor progression using soluble, biochemical markers. Cytokine profiling in blood or cerebrospinal fluid may also help with diagnosis and evaluating prognosis in cancer patients. While previous research has shown characterization of cytokine profiles for breast cancer, TCGA project is revealing how cytokines affect other tumors. For example, TCGA data showed that expression of high levels of miR-18 and low levels of *TGF-β* genes in the proneural glioblastoma subtype correlates with prolonged patient survival [73]. For the same subtype, proneural glioblastoma, increased levels of interferon/*STAT1* and genes related to interferon also determined poor survival outcome [74]. By incorporating key markers yielded from TCGA, cytokine profiling of tumors would provide an additional layer of diagnostic and prognostic information in conjunction with gene expression. Another forms of soluble biomarkers are those that undergo DNA methylation because this can be detected in blood serum samples of cancer patients [75]. Markers, such as O (6)-methylguanine-DNA methyltransferase (*MGMT*) that is known to confer resistance to temozolomide in glioblastomas, would help to predict a cancer patient’s personalized response to chemotherapy [76]. In a study by Majchrzak-Celińska *et al.*, methylation profiles of biomarkers—*MGMT, RASSF1A, p15INK4B,* and *p14ARF*—in cancer patient’s serum were found to match the methylation profiles in paired tumor samples in most cases [77]. In clinical practice, sequenced genomes of tumor profiles would direct which markers to test in biochemical assays. These assays can aid in the development of a personalized therapeutic plan and predict the effectiveness of various cancer drugs in a given individual.

The human metabolome may be another adjunct to link the gap between genotype and phenotype [78]. Integrating genetic data with metabolomics will add additional power to analysis, yielding improved accuracy and sensitivity. TCGA mutations can serve as a guide to focus metabolomic research, and the metabolomics can validate TCGA research by cross checking protein synthesis with DNA expression. Altered metabolism is a distinct feature of tumor cells, and it is known that specific genetic alterations, such as KRAS and BRAF, increase the expression of glucose transporter 1 [79,80]. Research in metabolomics has defined altered levels of metabolic enzymes and metabolites in various tumors, including oncogenic mutations that cause the malignancy [81,82]. It has been recently determined that the master transcriptional regulators of prostate cancer progression, AR and ETS gene fusions, control the regulatory enzymes of sarcosine, and therefore, high levels of sarcosine in the urine demonstrates a promising clinical biomarker of metastatic prostate disease [83]. TCGA-driven discovery may help elucidate the mechanisms of tumor-altered metabolism. This in turn may help develop quantitative, high-throughput metabolomics for systems biology to define metabolites as biomarkers for tumor progression. Key metabolites can be identified non-invasively and rapidly in the blood, cerebrospinal fluid, urine, saliva, and prostatic fluids. Another example of metastatic effects is the Warburg effect, which shows that under oxygen consuming (aerobic) conditions tumor tissues take up glucose and convert it to lactate ten-fold as much as than typical tissues in a given time [84]. TCGA may help us narrow down on some mutations tied in with cancer metabolism, opening up new targets for detection and treatment [85].

TCGA approach may shed new light on some not well-characterized cancer metastasis. For example, little was known about the metastasis of medulloblastoma, a tumor that is the most widely recognized childhood and adolescent tumor of the central nervous system (CNS). New data provide a mechanism by which metastasis of medulloblastoma yield a highly intrusive spread of tumor cells into the leptomeningeal space along the neuroaxis over the course of disease, this extraneural metastasis that is uncommon yet oftentimes deadly, happening in 1 to 5% of patients, the metastasis that presents near ventriculoperitoneal shunt [86]. Another example is the genomic characterization of 128 instances of metastases from the primary modalities of GBM defined the uncommonness of the metastasis based on histological and immunogenetic data [87]. Cancer genome information may therefore offer patients a hope to seek treatment for metastases to improve survival time.

### Therapy-driven cancer evolution may inspire TCGA-guided cancer prevention

The TCGA data may prove more significant in cancer prevention than in cancer diagnosis and treatment. This is an interesting concept as genomic heterogeneity within a single tumor evolves over time, location (dominate or dormant subclonal state that is regulated by tumor microenvironment), and random genetic drifting that serve as substrate for their evolution. An assumption that early diagnosis and early treatment yields the better results seems in question. Still, certain cancers have no effective treatment. In fact, cancer is curable only if the body’s immune system properly functions. Some mutations in our bodies may lead to cancer; the body’s immune system recognizes and eliminates the mutant cells. Current cancer treatments (radiation therapy, chemotherapy) have side effects on normal cells. The immune system is the hardest hit by these treatments and seems counterproductive to the goal [88]. However, specific data suggesting the relationship between the immune status and clinical response to molecularly targeted cancer therapy remains to be obtained. Currently limited data is available on pediatric patients’ immune response to chemotherapy [89] and on HER2-overexpressing breast cancer showing the relationship between markers of an antitumor immune response and clinical outcome [90].

Current therapeutic agents cannot exquisitely differentiate tumor from normal cells as both have similar signaling pathways [91]. In the future, individualized treatment depends on tumor behavior and maximizing recognition of abnormal cells by the patient’s immune system. Thus, an integrated and comprehensive treatment plan with cellular specificity will minimize side effects and maximize efficacy. To do that, we have to align precision diagnosis and treatment within an early therapeutic window [92] to make treatment simpler due to less tumor heterogeneity [49,93].

A mechanism by which cancer develops is by oncogenes accumulating and initiating unrestricted growth [94]. The mainstream treatment paradigm now is to use targeted drugs for cancer-causing genes or pathways. However, even for patients with deep sequencing of single cells, we look for targeted drugs, which is futile because the target is constantly changing [95]. That is because cancer cells are in constant evolution and transformation; their reactions to cancer drugs are also constantly changing [96]. So far this reality has not attracted the attention of cancer drug development - its drug screening is based on biopsy results obtained from patients 3–4 years previously and their derived xenografted tumors that are subjected to selection pressures of animal environment, different from human.

Robert Beckman thus conceives that cancer as a small ecological environment is in constant evolution and dynamic transformation. He thinks the application of mathematical models can determine the genetic evolution of cancer co-evolving with treatment [97]. That is particularly important for current treatments that focuses on quickly partitioned tumor cells, a way that actuates lethargic subclones of a tumor [49] and pressures tumor progression and metastasis [97,98]. Thus, TCGA approach may not lead to new cancer treatments, as it does not analyze real-time but rather post-diagnostic cancer specimens. This sampling allows for chasing after cancer mutations could help elucidate the history of tumor progression and its resultant heterogeneity. Such a history of cancer progression may guide the design of cancer prevention: a prevention timeline that shows a molecular cancer clock identifying the therapeutic window of surgical and chemical intervention [92], and a prevention profile that shows the onset of driver mutations and the switchboard signal of dormant to dominate subclone of a cancer evolving within its tumor Microenvironment. Its related non-cancer genetic background and its risk factors determine tumor microenvironment (Figure 3).

### Tracking subclonal behaviors with real-time imaging

How can we determine treatment-triggered tumor subclonal cell’s behavior in the changing microenvironment? Perhaps, an ex vivo tumor microenvironment of patient-derived tissues may be ideal to study the co-evolution of a tumor within a tumor environment [99] (Figure 2). Lack of knowledge on co-evolution dynamics potentially hinders the progress of a team-based, cross-disciplinary approach like TCGA [100]. Can these changes of cancer subclones be followed at the single cell level with a “biological global positioning system (bGPS)” [34], with visualization techniques for exploring oncogenomics data, allowing researchers to effectively visualize multidimensional oncogenomics datasets [101]? The idea of identifying one or a few simple biomarkers unique to a certain cancer type may prove to be unrealistic, as initially anticipated with the onset of TCGA. Biomarker panels initially demonstrating the needed 12 cancer-signaling pathways may differ in different stages of cancer because the number of cancer subclones will increase with time since onset of disease. Progress will need to be made to determine how these signaling pathways work in those cancer subclones.

Optical spectroscopic techniques like Raman spectroscopy have shown promising potential for in vivo pre-cancer and cancer diagnostics in a variety of organs [102]. Confocal micro-Raman spectroscopy--a valuable analytical tool in biological and medical field of research-allows probing molecular vibrations of samples without external labels or extensive preparation, ideal for real-time monitoring of tumor progression [103]. Raman spectroscopy can discriminate the differences found in regions characteristic for vibrations of carotenoids, unsaturated fatty acids, proteins, and interfacial water between the noncancerous and cancerous human breast tissues [104]. However, our practice led us to realize that a mega-database is needed to record the basic Raman spectrum for all the tissues, cells, and related molecules under the physiological and pathological conditions before we can translate information from Raman spectroscopy to clinical use. For example, the surface enhanced Raman scattering (SERS) technique shows the excellent specificity, high sensitivity (1 pg mL(−1)), as well as the great reproducibility of this SERS-conjugated immunoassay for simultaneous detection of tumor suppressor p53 and cyclin-dependent kinase inhibitor p21 for early cancer diagnosis [105]. The SERS-enabled narrow ‘fingerprint’ Raman spectra from the analyte molecules allows multiplex detection for biosensing applications [106], ideal for clinical use [107].

The challenge for imaging-guided evaluation of cancer-driven biomarker levels as an indicator for early cancer prediction is to correlate the immunoassay with the quantitative imaging technology [105]. First, we have to determine if TCGA-identified biomarkers are true biological drivers of cancer or background mutations that have no significance in the development or progression of a cancer. Information on genetic variations has been used to better understand the inheritance of and susceptibility to certain diseases, response to drugs, signaling pathways involved in normal versus disease states, and more. However, biological interpretation of thousands of variants is a bottleneck in extracting valuable insights from DNA sequencing studies, often requiring months of effort after completion of the reference genome alignment and variant calling steps. These limitations can largely be overcome by using more sophisticated informatics tools that can help interpret the biology accurately and in more detail.

### Cross-examination of big data sets helps decision-making

Accumulating profiles (pathology, microarrays, bulk tumor, and single cell genome sequencing - TCGA, mass spectrometry-based flow cytometry, imaging, therapy-driven genetic changes, and bioinformatics) is going forward to create a mega-database. This mega-database allows us to decipher cancer progression and treatment-driven evolution through super-computing to extract this information and synthesize new concepts [108]. Super-computing can interpret the large-scale, high-dimensional data sets that are generated by advanced technologies [109,110]. Lack of technology for integrating these different platforms might hinder the translation of big data into treatment. Conceptual integration of different diagnostic data remains to be determined. For example, how can MRI images integrate with morphological pathology, genome-scaled mutations, and biochemical assays in forming treatment strategy? How can we implement the automation in these processes? What is the accuracy of this automation? What is the cost for clinical applications?

In March 2012, the Obama Administration launched a $200 million “Big Data Research and Development Initiative”, which aimed to improve the tools and techniques needed to access, organize, and glean discoveries from huge volumes of digital data. The initiative would help to transform the use of big data in various sectors including scientific discovery and biomedical research. Big data in medical research is transforming and transitioning research from being hypothesis-driven to becoming data-driven. Efficient analysis and interpretation of big medical data can open up new avenues to explore, new questions to ask, and new ways to answer, leading to better understanding of diseases and development of better and personalized diagnostics and therapeutics [111]. However, such an assumption is based on fully understanding cancer initiation, progression, metastasis, subclonal switch for dormant cells, and evolving drug targets, which we know little about at the present. Thus, people should not be misled; no miracles happen from genome sequencing until we know how genes interact and evolve in the context of treatment. As geneticists Jon McClellan and Marie-Claire King stated, "a paradigm shift is that loci; previously thought of as origin of disease and as special targets are better thought of as associated with disease risk factors". In genetics, a rare variant of the common diseases may be the main cause of this conversion from a dormant state [112]; however, evolutionary forces introduce new variants to heterogeneity, leading to de novo temporal and spatial mutations in affected persons [113]. For these affected persons, their oncogenes alone are not sufficient to develop cancer, but with their non-cancer genetic background, may provide a supporting (promote cancer) microenvironment (Figure 3). As such, genomics should deal with a changing landscape of both cancer and non-cancer genetic background [114]; “Genomics is a way of doing science, not the way to do medicine” (Harold Varmus, the US National Cancer Institute). TCGA-generated cancer genome may serve as a common scaffold of information to accelerate cancer research. TCGA is a tool, which can be combined with single cell technology and bGPS [34] to follow how a cancer cell (dormant subclone) awakens, divides, grows to become a dominant cancer clone, and to take over the host over time. This progression process will give us the first handle as to when and where cancer starts and how we can attack it at the molecular and cellular level.

## Future perspectives

TCGA represented an unprecedented first step, a monumental effort to understand cancer. Here, we discuss current limitations of NGS ACTG genome projects, focusing on the problem of tumor heterogeneity and how this heterogeneity might inform the way we think about data from the large projects discussed here. The cancer genome is highly unstable and heterogeneous, making it difficult to differentiate driver mutations from passenger mutations. Large-scale unbiased genomic sequencing projects, such as TCGA, are ongoing for a large variety of clinical tumors to overcome the problem of intertumoral heterogeneity. As a result, recurrent somatic alterations have been identified by such projects, leading to the development of targeted therapies that have been shown to prolong some patient survival. The intratumoral heterogeneity has been a long-recognized issue, as correctly raised by recent reports.

TCGA is at a crossroads in the fight against cancer given the new understanding of intratumoral heterogeneity, requiring multi-spatial and multi-temporal sampling. We may have to perform single cell-based genome analysis, which will further complicate data interpretation and may increase price. We do not yet know what represents sufficient tumor area and the number of tumor cells that should be sampled to represent intratumoral heterogeneity. As TCGA builds the mega dataset of cancer gene sequences, we hope that TCGA can lead us to the “trunks” of diverging phylogenetic trees of cancer. These common points may be targets for therapeutics. Unless tumor heterogeneity is fully appreciated, we will lag behind the changes occurring within non-responsible clonal malignancies to form yet other types of clones.

Cancer appears not to be a single disease due to its innumerable genetic changes, each with its distinct molecular signature. Genetic mutations in cancer are inevitable and necessary to evolve under the selection pressure of the tumor microenvironment [98]. Practical approaches to these problems based on novel technologies (engineered tumor tissue graft models, single cell WGS) for massive parallel sequencing of DNA/RNA isolated from more homogeneous (microdissected) cancer cell population may redefine the TCGA project. With new research equipped with more high-tech studies (Single cells, bGPS) where we hope to follow the progression of cancer over time, TCGA may prove far more significant and more effective to pursue cancer prevention than cancer diagnosis and treatment, e.g., providing guided TCGA-defined catalogues of cancer risk factors. Specifically, cancer prevention can be realized with better understanding of how a therapy affects the genetic makeup of cancer over time in a clinical setting (e.g., engineered cancer tissue graft models, single cell technology, bGPS). Such study can validate mutations that arise during a tumor’s evolution under treatment and for which novel targeted therapies could be developed.
